# Case report: Slipped capital femoral epiphysis: a rare adverse event associated with FGFR tyrosine kinase inhibitor therapy in a child

**DOI:** 10.3389/fonc.2024.1399356

**Published:** 2024-05-24

**Authors:** Meziane Brizini, Tina Drimes, Catherine Bourne, Jessica Streilein, Annie Drapeau, Jens Wrogemann, Lori Anne Archer, Marc Del Bigio, Magimairajan Issai Vanan

**Affiliations:** ^1^ Division of Pediatric Hematology-Oncology, Cancer Care Manitoba, Winnipeg, MB, Canada; ^2^ Department of Pediatrics and Child Health, University of Manitoba, Winnipeg, MB, Canada; ^3^ Division of Nursing, Cancer Care Manitoba, Winnipeg, MB, Canada; ^4^ Division of Pharmacy, Cancer Care Manitoba, Winnipeg, MB, Canada; ^5^ Section of Neuro-Surgery, University of Manitoba, Winnipeg, MB, Canada; ^6^ Department of Radiology, University of Manitoba, Winnipeg, MB, Canada; ^7^ Section of Orthopedic Surgery, University of Manitoba, Winnipeg, MB, Canada; ^8^ Department of Pathology, University of Manitoba, Winnipeg, MB, Canada; ^9^ Paul Albrechtsen Research Institute, Cancer Care Manitoba, Winnipeg, MB, Canada

**Keywords:** fibroblast growth factor receptor inhibitors, Slipped capital femoral epiphysis, On-target skeletal toxicity, Erdafitinib, hypothalamic obesity

## Abstract

We report a case of slipped capital femoral epiphysis (SCFE), an on target skeletal toxicity of a pan-FGFR TKI inhibitor, erdafitinib. A 13-year-old boy was diagnosed to have an optic pathway/hypothalamic glioma with signs of increased intracranial pressure and obstructive hydrocephalus requiring placement of ventriculo-peritoneal (VP) shunt. Sequencing of the tumor showed FGFR1-tyrosine kinase domain internal tandem duplication (FGFR1-KD-ITD). He developed hypothalamic obesity with rapid weight gain and BMI >30. At 12 weeks of treatment with erdafitinib, he developed persistent knee pain. X-ray of the right hip showed SCFE. Erdafitinib was discontinued, and he underwent surgical pinning of the right hip. MRI at discontinuation of erdafitinib showed a 30% decrease in the size of the tumor, which has remained stable at 6 months follow-up. Our experience and literature review suggest that pediatric patients who are treated with pan-FGFR TKIs should be regularly monitored for skeletal side effects.

## Introduction

The cell surface receptor tyrosine kinases (RTKs) regulate fundamental cellular processes like cellular proliferation, differentiation, and survival through their signaling (1). Fibroblast growth factor receptor (FGFR) tyrosine kinase pathway signaling plays an important role in normal growth and development including metabolism and skeletal homeostasis (2). FGFR1–4 pathway alterations are rare in pediatric cancers (1%–3%) but are enriched in sarcomas and CNS tumors (3) where they account for 10% of pediatric low-grade gliomas and 4% of pediatric high-grade gliomas (4). FGFR tyrosine kinase inhibitors (FGFR-TKIs) are being increasingly used off-label in children with hard-to-treat tumors harboring specific FGFR alterations. This case report illustrates a rare but significant skeletal side effect of erdafitinib—a pan-FGFR inhibitor (2). Our case is an important addition to the recent literature (5) and informs pediatric oncologists to be monitoring for skeletal side effects when treating with FGFR-TKIs.

## Case summary

A previously healthy 13-year-old boy presented to the emergency department (ED) with 1 day history of emesis and headaches. His initial neurological exam was unremarkable. He progressively became unresponsive in the ED, showing signs of increased intracranial pressure with bradycardia, non-reactive pupils, and urinary incontinence. An urgent computerized tomography (CT) scan of the head showed a large suprasellar mass with mass effect on foramen of Munro bilaterally with obstructive hydrocephalus. The mass extended into the region of the Sylvian aqueduct resulting in CSF obstruction, which was relieved by placement of bilateral external ventricular drains (EVDs). On further query, the father reported that the patient has been symptomatic with intermittent headaches associated with nausea and vomiting for the past 6 months.

**Figure 1:**
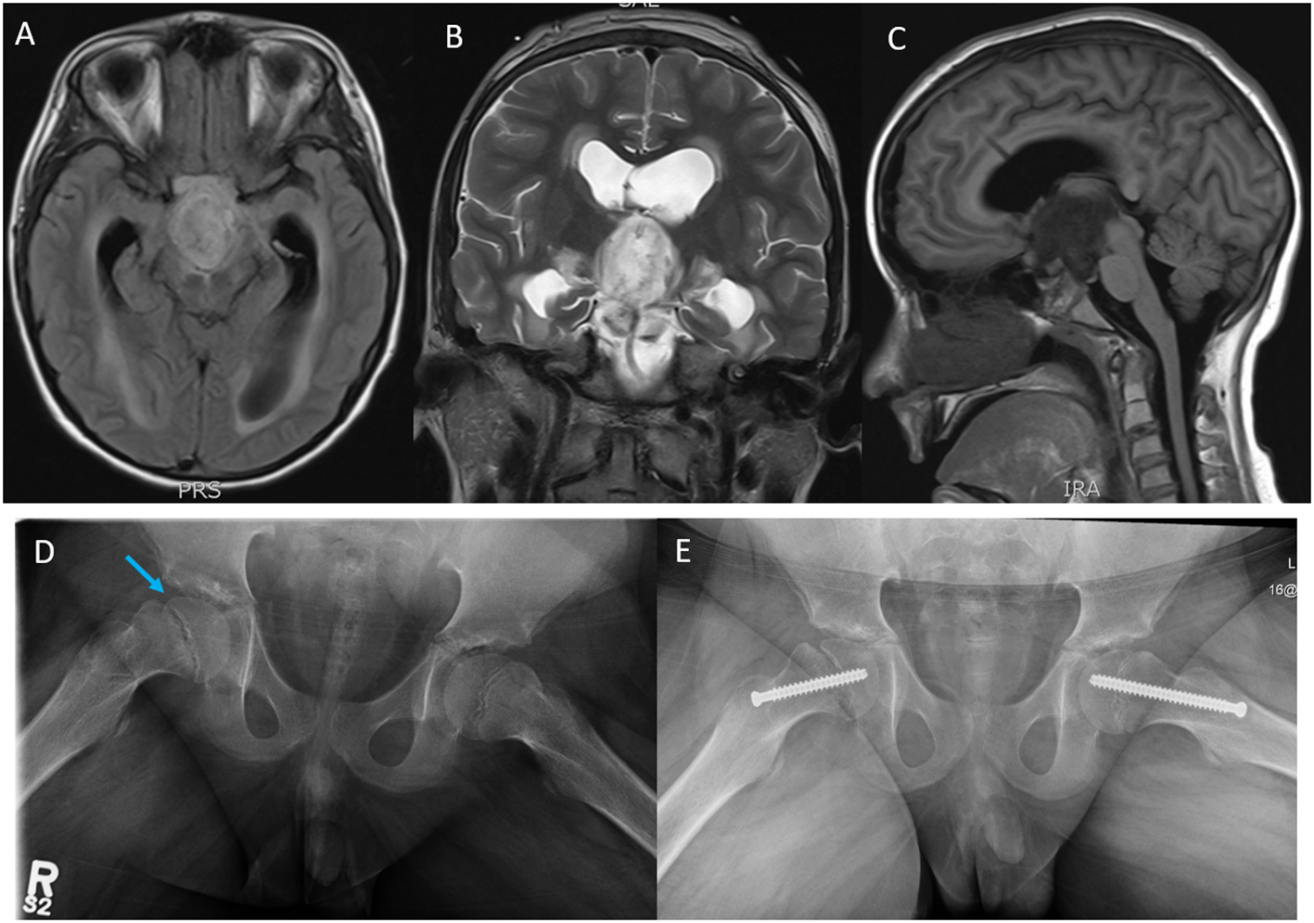
Magnetic resonance imaging (MRI); **(A)** axial T1 flair, **(B)** coronal T2, and **(C)** sagittal T1 flair images showing a rim-enhancing tumor mass likely arising from the Optic chiasm and extending into the third ventricle, the prepontine cistern, the interpeduncular cistern intermittent to the optic chiasm measuring 3.0 cm (AP) × 2.7 cm (CC) ×1.8 cm (T). **(D)** Plain X-ray films, open frog leg view showing inferior displacement of the right femoral capital epiphysis (blue arrow) relative to the femoral neck associated with widening of the proximal femoral growth plate and minimal joint effusion. **(E)** Surgical pinning to stabilize the capital epiphysis on the right hip and prophylactic pinning to prevent SCFE on the left hip.

After stabilization, baseline MRI showed rim-enhancing mass arising from the optic chiasm with mass effect on the pituitary infundibulum and perilesional edema involving the splayed cerebral peduncles and bilateral hypothalamus (**Figures 1A–C**). He was treated with Dexamethasone (1 mg, IV, QID) for increased intracranial pressure over 6 days and subsequently weaned off steroids over a week. Baseline investigations including renal/liver functions and endocrine tests including pituitary and thyroid functions tests were normal except for TSH of 0.3 mU/L (0.7–5.7 mU/L) with normal Free T3 and Free T4 values. The TSH values normalized in the subsequent follow-up visits. A ventriculo-peritoneal shunt was placed in the right lateral ventricle. Biopsy showed a histologically low-grade astrocytoma (WHO, Grade 1). TruSight RNA Pan-Cancer NGS panel showed FGFR1-tyrosine kinase domain internal tandem duplication (FGFR1-KD-ITD) (chromosome 8, exon 18–exon 10). The tumor was negative for BRAF mutation/fusion. Two weeks after biopsy, he presented to the ED with 1-week history of abdominal pain and was diagnosed with abdominal abscess due to VP shunt infection. The shunt was removed. CSF cultures grew *Staphylococcus aureus* (methicillin sensitive, MSSA). He was subsequently treated with IV antibiotics for 6 weeks.

**Figure 2:**
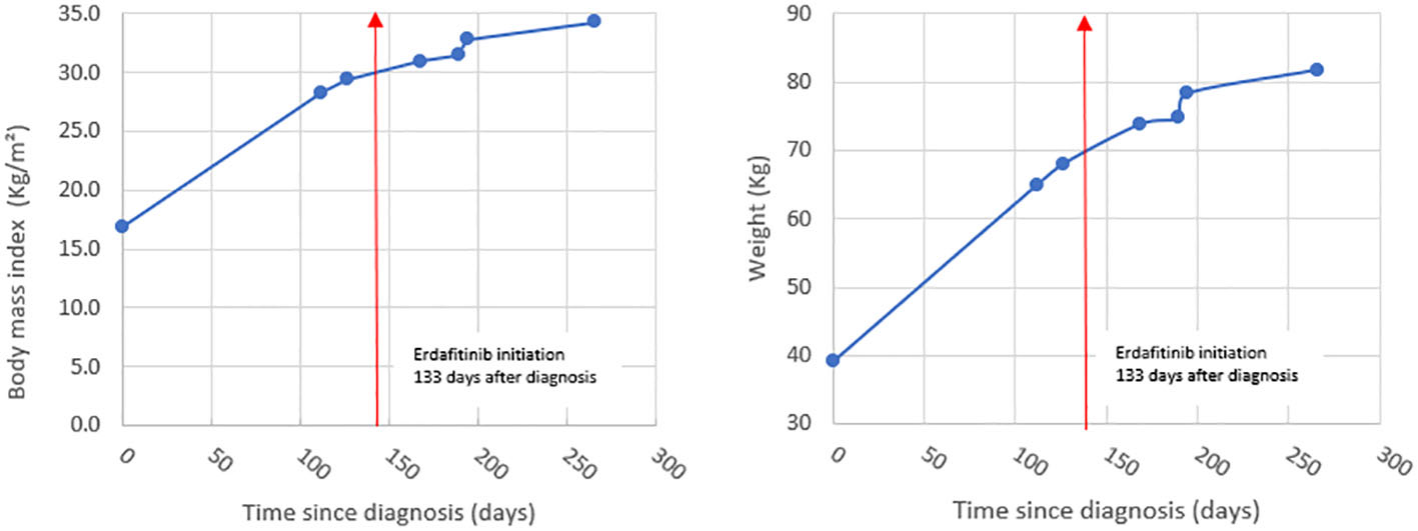
Graphs showing BMI and rate of weight gain in our patient.

Our patient developed hypothalamic obesity due to the location of the tumor and the perilesional edema involving the hypothalamus at presentation (**Figure 1C**). This was clinically manifested by a voracious insatiable appetite and accelerated rate of weight gain (82.1 kg->97% percentile for age and sex) (**Figure 2**). After a 4-month delay from the initial diagnosis, he was started on erdafitinib 4.7mg/m^2^/day (Janssen BioAdvance), a pan-FGFR1–4 inhibitor, through a special access program. Four weeks later, he developed nail side effects (discoloration and brittleness) and hyperphosphatemia requiring chelation. Erdafitinib was withheld for a week and restarted as per protocol. At approximately 7 weeks into therapy, he started complaining of intermittent pain in his right leg (knee and ankles), progressing to limping, difficult weight bearing, and complete cessation of ambulation. There was no history of trauma or past history of knee, leg, or hip pain. X-rays of the knee/ankles were reported as normal. At approximately 12 weeks of treatment, the persistent knee pain was suspected to be due to pathology of the hip with referred pain to the knee. X-ray of the hips (**Figure 1D**) showed slipped capital femoral epiphysis (SCFE) of the right hip. Erdafitinib was discontinued, and he underwent surgery with *in situ* pinning of the right hip and prophylactic pinning of the left hip (to prevent SCFE) (**Figure 1E**). MRI of the brain at the time of discontinuation of the medication showed a 30% decrease in the size of the tumor, which has remained stable on two subsequent follow-up MRIs.

## Discussion

FGFR1–4 inhibitors are being frequently used for hard-to-treat pediatric cancers with FGFR alterations in both ongoing clinical trials (NCI-COG-Pediatric MATCH trial, Phase II, NCT03155620) (6) and off-label (3, 7). FGFR1 and FGFR3 signaling negatively regulates endochondral bone growth by inhibiting growth plate chondrocytes by suppressing mitogenic activity (8, 9). Inhibition of these receptors causes increased linear growth velocity in growing/immature skeleton and predisposes to bone- and joint-related complications (5). These complications are rare in adults, as their growth plates are fused resulting in minimal FGFR signaling in growth plate chondrocytes.

Tyrosine kinase inhibitors are increasingly being used in the treatment of pediatric cancers, and TKI-induced adverse effects have been frequently reported involving multiple organs, most commonly skin, gastrointestinal tract, blood, and cardiac side effects (10). Skeletal side effects of targeted therapies (TKIs, biological therapies) like osteonecrosis and fractures are summarized in a recent review by Konarski et al. (11). SCFE and other skeletal side effects as a complication of FGFR-selective inhibitors was recently reported by Sait et al. (5). In their series, all the three pediatric patients with SCFE had increased growth velocity (**Table 1**). Two of the three patients were also reported to have obesity. We did not see increased growth velocity in our patient (height between 25th and 50th percentile for age and sex). This could be probably explained by the short duration of erdafitinib treatment in our patient (12 weeks). The unfortunate delay in starting erdafitinib due to MSSA infection resulted in our patient being obese (BMI> 30, >99th percentile for age and sex) at the start of the drug. Obesity has been shown to be a strong and validated risk factor for SCFE (12, 13). Body Mass Index (BMI) >95th percentile for age and sex has a strong correlation with SCFE when compared to children with BMI <85th percentile for age and sex (14). Based on the rate of weight gain and the BMI, we decided to do prophylactic *in situ* pinning for the contralateral (left) hip to prevent SCFE (14).

**Table 1:** Characteristics of patients with SCFE treated with FGFR TKIs.

Patient number/age (yrs)/sex	Tumor histology/site	FGFR alteration	Skeletal AEs	Non-skeletal AEs	DOT (months)	FGFRi
1) 8/F	Pilomyxoid astrocytoma/Optic pathway	FGFR1 mutation (V592M, K687E)	SCFE (b/l hips), AVN of left hip, non-traumatic fractures (finger/hip)	Hyperphosphatemia	9	Debio1347
2) 14/M	Rosette-forming low-grade glioneuronal tumor/cerebellum	FGFR3–TACC3 fusion	SCFE (right hip), osteochondritis dissecans (b/l), coxa valga deformity (b/l)	Hyperphosphatemia	40	Debio1347
3) 12/M	Diffuse brainstem glioma/brainstem	FGFR2–VPS35 fusion	SCFE (right hip), non-traumatic fractures (b/l tibia, vertebral compression fractures)	Hyperphosphatemia	5	Erdafitinib
4) 13/M	Low-grade astrocytoma/Optic pathway (chiasm)	FGFR1-TKD-ITD (exons 18,10)	SCFE (right hip)	Onycholysis (Grade III), hyperphosphatemia	3	Erdafitinib

AEs, Adverse events; AVN, avascular necrosis; DOT, duration of therapy; F, female; FGFR, fibroblast growth factor receptor; FGFRi, FGFR inhibitor; M, male; SCFE, slipped capital femoral epiphysis; Yrs, years; TACC, transforming acidic coiled coil; b/l, bilateral.

The first three patients are from Ref. 5.

Pan-FGFR1–4 inhibitors (like erdafitinib) are pharmacologically more potent when compared to FGFR1/2/3 inhibitors (like Debio1347): IC50 values (in nanomolar, nM) for erdafitinib and Debio1347 are as follows: FGFR1 (2.0 nM vs. 9.3 nM), FGFR2 (2.0 nM vs. 7.6 nM), and FGFR3 (4.0 nM vs. 22 nM) (15). The increased potency of erdafitinib in combination with other risk factors like obesity and hyperphosphatemia could probably explain the relatively early onset of SCFE in patients treated with Pan-FGFR1–4 inhibitor erdafitinib when compared to FGFR1/2/3 inhibitor Debio1347 (**Table 1**). The dose of erdafitinib for both patients in this series was based on the recommendations of the NCI-COG Pediatric MATCH, APEC1621B protocol, which is 4.7 mg/m^2^/day (8 mg/1.7 m^2^) up to a maximum daily dose of 8 mg, orally, once daily (adult recommended phase II dose-RP2D adjusted for BSA).

Hyperphosphatemia, another on-target toxicity specific to FGFR TKIs, was seen in all the patients with SCFE (**Table 1**). Hyperphosphatemia can potentially contribute to the SCFE, as it can lead to increased bone turnover and bone fragility (16). Our patient did not receive any chemotherapy or radiation prior to starting erdafitinib. Patient 1 in the series was previously treated with chemotherapy (7), and this could have contributed to the SCFE in this patient (17).

## Summary

We report a case of SCFE, an on-target skeletal toxicity of a pan-FGFR TKI inhibitor, erdafitinib. Central hypothalamic obesity, secondary to the location of the tumor along with hyperphosphatemia due to drug side effect, contributed to the accelerated onset of SCFE in our patient. Based on our experience and literature review (5) of pediatric patients who are treated with pan-FGFR TKIs, we recommend the following: a) monitor both rate of weight gain/BMI for obesity (especially in suprasellar tumors) and growth velocity while on therapy; b) promptly rule out on-target skeletal toxicities (fractures, SCFE, and osteochondritis) at the onset of clinical symptoms (knee, thigh, or groin pain) with appropriate diagnostic imaging (AP/frog leg lateral views of hip and knee X-rays); c) regular surveillance imaging of the hips to monitor for SCFE especially in pediatric patients with increased weight gain (suprasellar tumors) and/or growth velocity; and d) update the drug toxicity profile of FGFR TKIs to include skeletal toxicities and inform parents/patients of these toxicities at the time of consent for therapy.

## Data availability statement

The original contributions presented in the study are included in the article/Supplementary Material. Further inquiries can be directed to the corresponding author.

## Ethics statement

Written informed consent was obtained from the individual(s) for the publication of any potentially identifiable images or data included in this article.

## Author contributions

MB: Conceptualization, Data curation, Formal analysis, Investigation, Writing – original draft. TD: Investigation, Resources, Writing – review & editing. CB: Investigation, Resources, Writing – review & editing. JS: Investigation, Resources, Writing – review & editing. AD: Conceptualization, Data curation, Investigation, Resources, Validation, Writing – review & editing. JW: Data curation, Resources, Validation, Writing – review & editing. LA: Data curation, Investigation, Resources, Validation, Writing – review & editing. MD: Data curation, Investigation, Resources, Validation, Writing – review & editing. MV: Conceptualization, Data curation, Investigation, Resources, Supervision, Validation, Writing – original draft, Writing – review & editing.
